# Exosome derived from CD137‐modified endothelial cells regulates the Th17 responses in atherosclerosis

**DOI:** 10.1111/jcmm.15130

**Published:** 2020-03-09

**Authors:** Liangjie Xu, Tianxin Geng, Guangyao Zang, Li Bo, Yi Liang, Hong Zhou, Jinchuan Yan

**Affiliations:** ^1^ Department of Cardiology Affiliated Hospital of Jiangsu University Zhenjiang China; ^2^ School of Medicine Jiangsu University Zhenjiang China

**Keywords:** atherosclerosis, endothelial cells; Th17 cells, exosomes

## Abstract

The role of exosomes derived from endothelial cells (ECs) in the progression of atherosclerosis (AS) and inflammation remains largely unexplored. We aimed to investigate whether exosome derived from CD137‐modified ECs (CD137‐Exo) played a major role in AS and to elucidate the potential mechanism underlying the inflammatory effect. Exosomes derived from mouse brain microvascular ECs treated with agonist anti‐CD137 antibody were used to explore the effect of CD137 signalling in AS and inflammation in vitro and *vivo*. CD137‐Exo efficiently induced the progression of AS in ApoE^−/−^ mice. CD137‐Exo increased the proportion of Th17 cells both in vitro and *vivo*. The IL‐6 contained in CD137‐Exo which is regulated by Akt and NF‐КB pathway was verified to activate Th17 cell differentiation. IL‐17 increased apoptosis, inhibited cell viability and improved lactate dehydrogenase (LDH) release in ECs subjected to inflammation induced by lipopolysaccharide (LPS). The expression of soluble intercellular adhesion molecule1 (sICAM‐1), monocyte chemoattractant protein‐1 (MCP‐1) and E‐selectin in the supernatants of ECs after IL‐17 treatment was dramatically increased. CD137‐Exo promoted the progression of AS and Th17 cell differentiation *via* NF‐КB pathway mediated IL‐6 expression. This finding provided a potential method to prevent local and peripheral inflammation in AS.

## 1 INTRODUCTION

T helpers 17 (Th17) cells have been identified as a new subset of lymphocytes which are shown to promote atherosclerosis (AS), and their associated cytokines in the pathogenesis of AS appear to be contradictory at first glance.^1^, ^2^, ^3^ Recently, growing attention has been paid to the relative contribution of the local *vs*. peripheral inflammation to the procession of AS. Here, we focused on the communication between peripheral inflammation and vascular inflammation of Th17 cell differentiation during AS.

Exosomes are a subset of extracellular vesicles (EVs) released by almost all types of cells, and they play a significant role in cellular proliferation, apoptosis, stress response and differentiation in the initial stage and development of AS.^4^, ^5^ In the present study, we investigated exosome‐mediated cell‐to‐cell communication, which induced Th17 cell differentiation and promoted progression of the atherosclerotic plaque.

In our previous studies, CD137‐CD137L signalling has been shown to be involved in AS.^6^ We have also demonstrated that increased CD137 level may contribute to the instability of atherosclerotic lesion. Endothelial cells (ECs) located in the intima layer are in direct contact with the lumen.^7^ ECs can be activated by various factors, among which a number of pro‐inflammatory cytokines are produced by immunocytes in blood, including interleukin (IL)‐6, IL‐12 and tumour necrosis factor (TNF).^8^, ^9^ Helped with the ECs‐derived exosomes, peripheral inflammation and vascular inflammation are triggered, and then, the dysfunction of ECs is amplified, promoting atherosclerotic process to the next step. Therefore, we hypothesized that the changes of the blood vessels triggered a local endothelial inflammation via activating of CD137 signalling and inducing the EC dysfunction. Exosomes derived from ECs mediate cell‐to‐cell communication, and crosstalk between organs further promotes peripheral inflammatory responses, which induces Th17 cell differentiation to promote progression of the atherosclerotic plaque.

In the present study, we hypothesized that the CD137‐CD137L signalling induced the formation of atherosclerotic plaque via regulating the Th17 cell responses through modulating EC‐derived exosomes. The results showed that CD137‐CD137L signalling regulated EC‐derived exosomes to induce Th17 cell differentiation through IL‐6 and promoted AS progression in ApoE^−/−^ mouse. Therefore, our findings implied that EC‐derived exosomes could be used as a new cell therapy of AS.

## 2 MATERIALS AND METHODS

### 2.1 Animals

ApoE^−/−^ mice aged eight weeks were used in this study. The protocol was approved by the Animal Care and Use Committee of the Affiliated Hospital of Jiangsu University, China and conformed to the Guide for the Care and Use of Laboratory Animals published by the National Institutes of Health (NIH publication no.85‐23, revise 1996).

Twenty micro grams exosomes were induced with or without co‐cultured with siRNA IL‐6 were injected weekly to 8‐week‐old male mice for 12 weeks. The mice of control group were injected with PBS. Mice were divided into five groups: (a) control; (b) con‐EXO; (c) CD137‐EXO; (d) siR IL‐6; and (e) siR‐NC. The mice at the age of 20 weeks were killed and removed the aortas, spleen from connective tissue.

### 2.2 EC culture

To determine whether CD137‐CD137 signalling regulated EC‐derived exosomes to induce Th17 cells responses through IL‐6, we activated the CD137 signalling with agonist anti‐CD137 antibody (10 μg/mL, Sangon Biotech) in the absence or presence of IL‐6 siRNA to induce exosomes. EC‐derived exosomes were divided into four groups as follows: (a) con‐Exo: ECs were cultured in the absence of agonist anti‐CD137 antibody; (b) CD137‐Exo: ECs were cultured in the presence of agonist anti‐CD137 antibody; (c) si IL‐6: ECs were cultured in the presence of agonist anti‐CD137 antibody and IL‐6 siRNA; and (d) si‐NC: ECs were cultured in the presence of agonist anti‐CD137 antibody and siRNA NC.

### 2.3 Co‐culture of exosomes and T cells

Splenic CD4^+^T cells were isolated from wild‐type mice (6‐8 weeks old) using CD4^+^T‐cell microbeads (Miltenyi Biotec). For Th17 cell differentiation,^10^ CD4^+^T cells were cultured for 72 hours in the presence of anti‐CD28 mAb (2 μg/mL), IL‐6 (30 ng/mL), TGF‐β(5 ng/mL), anti‐IFN‐γmAb (5 μg/mL), anti‐IL‐4 mAb (5 μg/mL) and IL‐23 (30 ng/mL) in a 24‐well plate pre‐coated with anti‐CD3 mAb (2 μg/mL).

EC‐derived exosomes regulate Th17 cell differentiation in vitro were assigned to the following five groups: (a) control; (b) con‐Exo; (c) CD137‐Exo; (d) si IL‐6; and (e) si‐NC.

### 2.4 Isolation, analysis and labelling of exosomes

Serum‐free conditioned medium was collected after treatment of ECs. Exosomes were also isolated with the ExoQuick Plasma prep and the Exosomes Precipitation Kit according to the manufacturer's instructions (System Biosciences).^11^, ^12^, ^13^

The size distribution and ultrastructure of exosomes were analysed by NanoSight and transmission electron microscopy, respectively. Protein markers, including CD81, CD9 and calnexin, were determined by Western blotting analysis.

For uptake studies, purified exosomes were labelled with a PKH‐26 (Life Technologies) kit.

### 2.5 Immunofluorescence microscopy

For immunofluorescence staining, the sections were incubated with primary antibody: CD4 (RD, BNP2‐25191), IL‐17(Abcam, ab79056) overnight, followed by secondary antibody.

### 2.6 Flow cytometric analysis

The cell suspensions from spleen were stained with anti‐CD4 mAbs (eBioscience) followed by permeabilization and incubation with intracellular anti‐IL‐17 mAbs (eBioscience). The stained cells were analysed with a FACSCalibur instrument (Becton Dickinson).

### 2.7 Enzyme‐linked immunosorbent assay

The levels of IL‐6, sICAM‐1 MCP‐1 and E‐selectin from supernatant were measured using a commercial Biotin/Avidin based Sandwich ELISA (eBiosciences).

### 2.8 Quantitative real‐time PCR (qRT‐PCR)

Total RNA was isolated and reverse‐transcribed using the ReverTra ACE qPCR RT Kit (TOYOBO, Japan) according to the manufacturer's instructions. The following primers were sequences utilized: IL‐6, 5′‐ CTGCAAGAGACTTCCATCCAG‐3′ (forward), 5′‐ AGTGGTATAGACAGGTCTGTTGG‐3′ (reverse); β‐actin, 5′‐ CGGTCAGGTCATCACTATCG‐3′ (forward), 5′‐ TTCCATACCCAGGAAGGAAG‐3′ (reverse); IL‐12: P: 5′‐ TGACATGGTGAAGACGGC‐3′ (forward), 5′‐ GCCTGGAACTCTGTCTGGTA‐3′ (reverse); IL‐23:5′‐ CAGCAGCTCTCTCGGAATCTC‐3′ (forward), 5′‐ TGGATACGGGGCACATTATTTTT‐3′ (reverse); IL‐1β: 5′‐ GAAATGCCACCTTTTGACAGTG‐3′ (forward), 5′‐ TGGATGCTCTCATCAGGACAG‐3′ (reverse).

### 2.9 Small‐interfering (si) RNA experiment in CD4^+^T cells

IL‐6 siRNA was obtained from Guangzhou RiboBio. There sequences were as follows: CCAAGACCATCCAAATTCAT. siRNA was prepared according to the transfection protocol for cell culture.

### 2.10 Statistical analysis

All values are presented as the mean ± SD. Results were analysed by an unpaired, two‐tailed Student's t test (two groups) or ANOVA (three or more groups) as appropriate. All the analyses were performed using SPSS software (11.5). *P* < .05 was considered to indicate statistical significance.

## 3 RESULTS

### 3.1 Successful isolation of exosomes from culture medium of ECs

**Figure 1:**
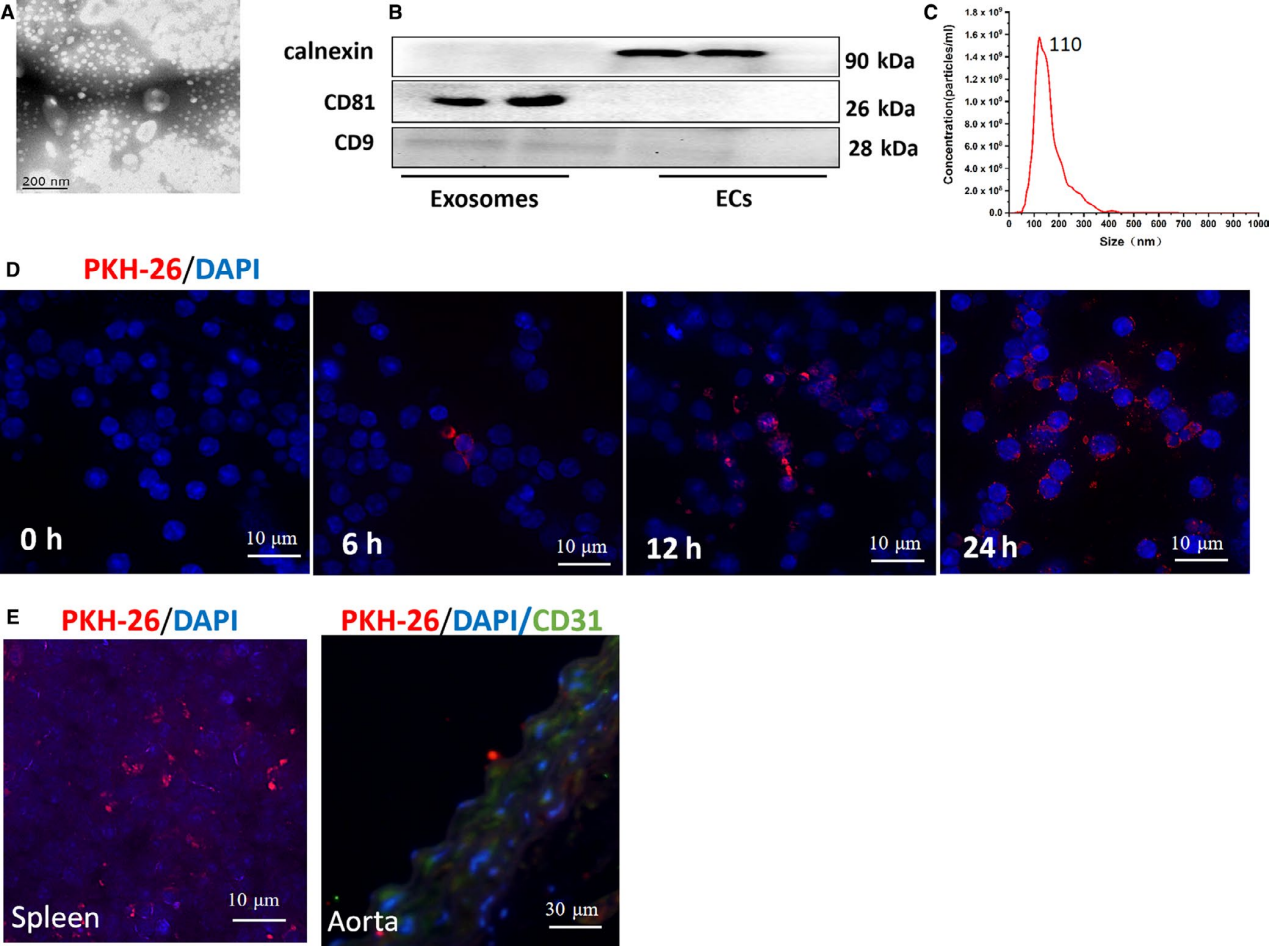
Successful isolation of exosomes from ECs culture medium and the uptake assay of exosomes in vitro and vivo. A, The ultrastructure of exosomes by transmission electron microscopy. Bar: 200 nm. B, Expression of exosomes markers, CD81, CD9 and calnexin. C, The size distribution profile of exosomes by NanoSight. D, The uptake of PKH26‐labelled ECs‐derived exosomes (red) by splenic CD4^+^T cells in vitro. Nuclei were stained with DAPI (blue). Bar: 10 μm. E, The uptake of exosomes by spleen (Bar: 10 μm) and aorta (Bar: 30 μm) in vivo. Nuclei were stained with DAPI (blue). The aortas were stained with CD31 (green) and DAPI (blue)

The ultrastructure of exosomes was analysed by using transmission electron microscopy and NanoSight (Figure 1A,B). The results showed a cup‐shaped morphology of 30‐150 nm in size, reaching a peak size of 110 nm (Figure 1C). The expressions of exosomal markers, CD81 and CD9, as well as the negative marker for exosomes, Calnexin were confirmed by Western blotting analysis (Figure 1D). To examine whether CD4^+^T cells could uptake exosomes, we labelled exosomes with a red fluorescent marker, PKH26, and incubated the exosomes with CD4^+^T cells. The results showed that CD4^+^T cells could uptake exosomes within 6 hours, and more exosomes were further up‐taken within 24 hours. Next, we intravenously injected PKH26‐labelled exosomes into ApoE^−/−^ mice and found that exosomes could be up‐taken by spleen and aorta ECs (Figure 1E).

### 3.2 CD137 signalling regulates Th17 cells through modulating IL‐6 of EC‐derived exosomes in vitro

**Figure 2:**
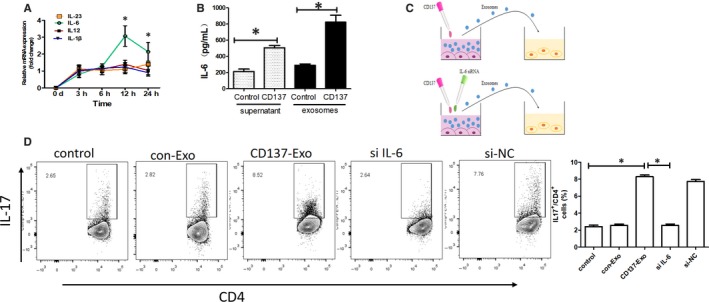
CD137 signalling regulated Th17 cells through modulating IL‐6 of ECs‐derived exosomes in vitro. A, IL‐6, IL‐12, IL‐23 and IL‐1β mRNA levels in ECs were measured with qRT‐PCR analysis (*P < .05, n = 3). B, IL‐6 concentration in supernatant and exosomes was measured with ELISA analysis (*P < .05, n = 5). C, ECs were transfected with siRNA IL‐6 or siRNA NC. Then, exosomes were isolated from transfected ECs and incubated with Th17 cell differentiation system. D, Flow cytometric analysis of Th17 cells (*P < .05, n = 5)

Because chronic inflammation plays an important role in AS,^14^, ^15^ we selected four pro‐inflammatory cytokines (IL‐6, IL‐12, IL‐23 and IL‐1β) in our investigation, which are involved in AS. Using qRT‐PCR and ELISA, we observed that the expression of IL‐6 in exosomes was significantly increased after activation of CD137 signalling (Figure 2A,B).

Studies have shown that IL‐6 signalling in CD4 + T cells facilitates Th17 cell development.^16^ To determine whether CD137 regulated EC‐derived exosomes to promote Th17 cell differentiation through IL‐6 signalling, we depleted IL‐6 in EC‐derived exosomes (Figure 2C). We observed that the proportion of Th17 cells was increased compared with the control and con‐Exo groups upon CD137‐Exo treatment. In contrast, the siRNA IL6‐EXO treatment significantly decreased the proportion of the Th17 cells (Figure 2D).

### 3.3 CD137 signalling regulates Th17 cells through modulating IL‐6 of EC‐derived exosomes in vivo

**Figure 3:**
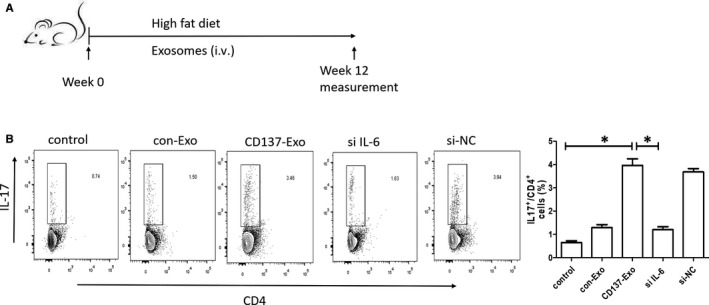
CD137 signalling regulated Th17 cells through modulating IL‐6 of ECs‐derived exosomes in vivo. A, Overview of the in vivo experimental procedure. B, Flow cytometric analysis of Th17 cells (*P < .05, n = 5)

After weekly injection of exosomes for a period of 12 weeks (Figure 3A), we demonstrated that the CD137‐EXO group had a larger plaque size compared with the control and the con‐Exo groups, while the si IL‐6 group showed a smaller plaque size compared with the control and the si‐NC groups (Figure 3B).

### 3.4 CD137 signalling promotes the plaque inflammation by enhancing of Th17 cell responses

**Figure 4:**
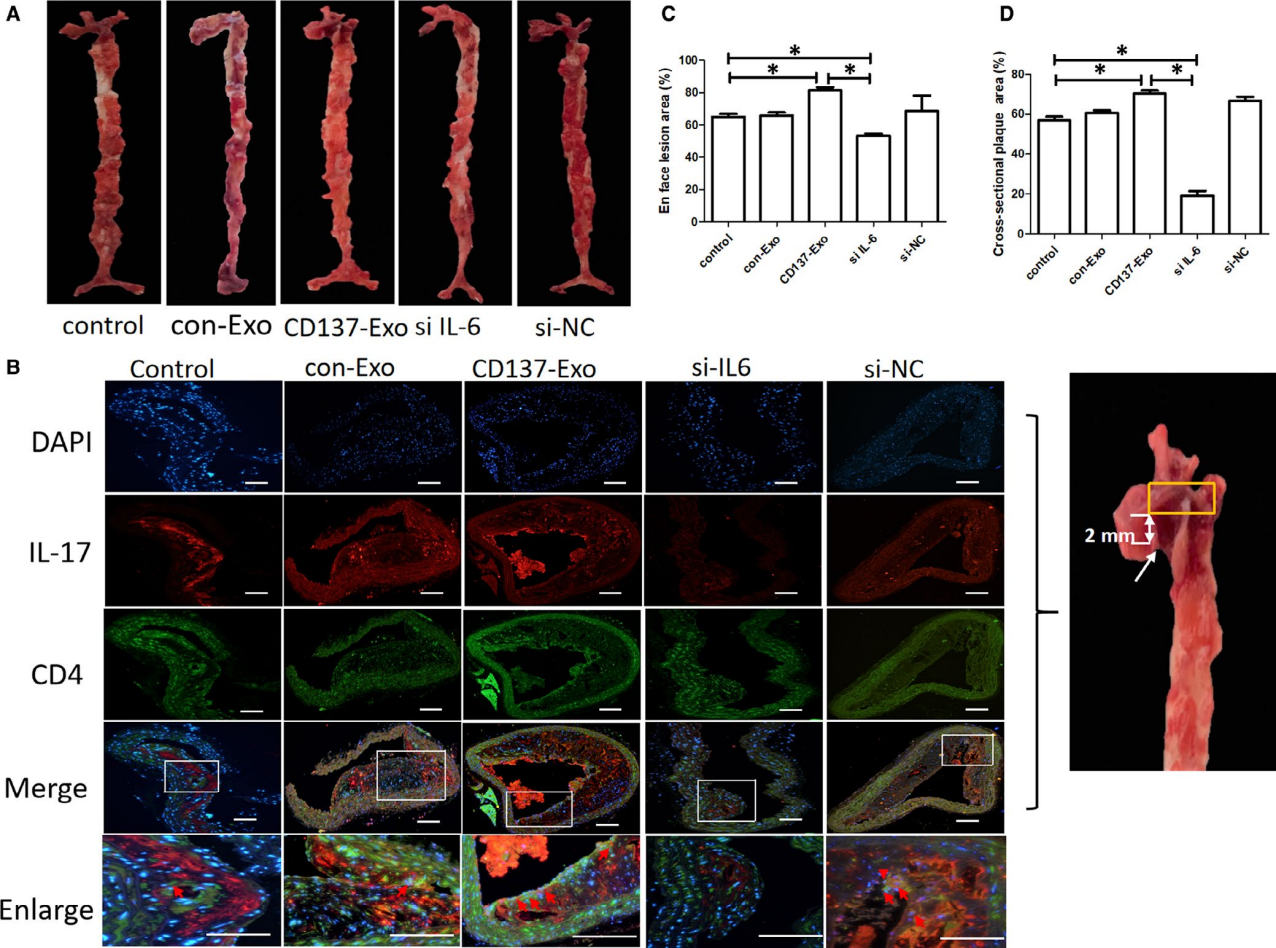
CD137 signalling induced Th17 cell differentiation and atherosclerosis through modulating IL‐6 of ECs‐derived exosomes in ApoE^−/−^ mice. A, Representative en face Oil red O staining of the entire aortas of the five groups of ApoE^−/−^ mice. B, Representative cross‐sectional images of the aortic plaque of the five groups of ApoE^−/−^ mice. The cross‐sectional slice of the aorta (yellow box area), 2 mm above the aortic valve (white arrow). The expression of Th17 cells was stained by immunofluorescence analysis. Bar: 200 μm. C, Quantification of the en face lesion areas of the entire aortas of the five groups of ApoE^−/−^ mice (*P < .05, n = 3). D, Quantification of the cross‐sectional plaque areas of the five group of ApoE^−/−^ mice (*P < .05, n = 3)

From the above‐mentioned data, we hypothesized that the CD137‐CD137L signalling induced the formation of atherosclerotic plaque via regulating the differentiation of Th17 cells through modulating EC‐derived exosomes. We cultured ECs in the presence of agonist anti‐CD137 antibody and IL‐6 siRNA, red oil staining and immunofluorescence revealed that the proportion of Th17 cells was significantly increased in the plaque of CD137‐Exo group compared with the control group. In contrast, the proportion of Th17 cells was markedly decreased in the plaque of si IL‐6 group compared with the CD137‐Exo group (Figure 4A‐D).

### 3.5 Akt and NF‐КB mediate IL6 induction by CD137

**Figure 5:**
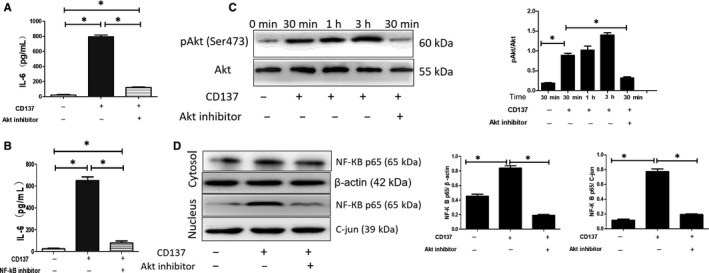
CD137 signalling regulated IL‐6 expression in ECs through Akt and NF‐КB pathway. A, CD137‐provoked up‐regulation of IL‐6 is blocked by Akt inhibitor (*P < .05, n = 5). B, CD137‐provoked up‐regulation of IL‐6 is blocked by NF‐КB inhibitor (*P < .05, n = 5). C, CD137 increased expression of Akt at serine 473 after 30 min of incubation, whereas attenuated by Akt inhibitor (*P < .05, n = 3). D, CD137‐induced expression of nuclear NF‐КB p65 is attenuated by Akt inhibitor (*P < .05, n = 3)

First, we activated the CD137 signalling with agonist anti‐CD137 antibody. We found that the expressions of pAkt (Ser473) and NF‐КB p65 were significantly increased after the exposure to agonist anti‐CD137 antibody. Figure 5C shows that the expression of pAkt (Ser473) was gradually increased in a time‐dependent manner, and it reached the peak at 3 hours after exposure to agonist anti‐CD137 antibody. We inhibited the Akt activity by the specific inhibitor, AktI. We found that the CD137L‐induced expression of Akt at serine 473 could be significantly attenuated by AktI. Furthermore, CD137L‐induced up‐regulation of IL‐6 could be blocked by AktI (Figure 5A). Similarly, we inhibited the NF‐КB activity by the specific inhibitor, PDTC. We found that CD137‐induced NF‐КB p65 translocation into the nucleus could be significantly attenuated by AktI (Figure 2D). In addition, CD137‐induced up‐regulation of IL‐6 could be blocked by PDTC (Figure 5B).

### 3.6 IL‐17 stimulates EC dysfunction upon lipopolysaccharide (LPS) exposure

**Figure 6:**
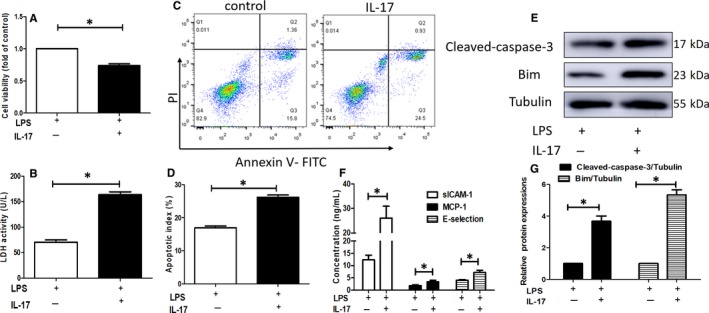
IL‐17 stimulates ECs dysfunction upon lipopolysaccharide (LPS) exposure. A‐D, IL‐17 inhibited cell viability (A) (*P < .05, n = 5), improved lactate dehydrogenase (LDH) release (B) (*P < .05, n = 5) and increased apoptosis in ECs subjected to inflammation induced by LPS (C, D) (*P < .05, n = 3). E and G, Western blot analysis of the expression of cleaved caspase‐3 and Bim (*P < .05, n = 3). F, The production of chemokine and adhesive molecules were determined by ELISA (*P < .05, n = 3)

We next assessed whether the increased proportion of Th17 cells, which mainly secrete IL‐17, also played a crucial role in EC dysfunction in an inflammatory environment. Since it is difficult to achieve the co‐culture of Th17 cells and ECs, we explored the effect of IL‐17 on EC functions, such as cell proliferation, viability and apoptosis. Figure 6A‐D illustrates that IL‐17 increased apoptosis, inhibited cell viability and improved lactate dehydrogenase (LDH) release in ECs upon LPS challenge. Western blotting analysis revealed that IL‐17 induced the expressions of pro‐apoptosis genes (Bim and cleaved caspase‐3) (Figure 6E,G).

We found that the expressions of sICAM‐1, MCP‐1 and E‐selectin were dramatically increased in the supernatants of ECs after IL‐17 treatment (Figure 6F). These data suggested that IL‐17 could activate ECs and contribute to endothelial dysfunction.

## 4 DISCUSSION

In this study, we achieved three major findings. First, exosome derived from CD137‐modified ECs regulated the Th17 cell response in AS development. Second, the expression of IL‐6 in exosomes derived from ECs was significantly increased after activation of CD137 signalling, and IL‐6 in EC‐derived exosomes potentially played an importantly role in AS development by prompting Th17 cell responses through the up‐regulation of AKT/ NF‐КB signalling.

Emerging evidence has revealed that CD137‐CD137L signalling plays an important role in the plaque inflammation during AS development.^4^, ^5^, ^6^ However, it remains unclear whether EC‐derived exosomes participate in the Th17 cell differentiation, and if so, whether CD137‐CD137L signalling can regulate the immunoregulatory properties in AS development. Therefore, we aimed to investigate the molecular mechanism underlying the regulation of exosomes derived from CD137‐modified ECs on the Th17 cell responses in AS development.

In this study, we first observed that EC‐derived exosomes induced inflammatory responses of AS by prompting Th17 cell differentiation after activation of CD137 signalling. A wealth of evidence has been reported that IL‐6 plays a crucial role in the Th17 cell differentiation in AS.^17^, ^18^ Our recent study has shown that CD137‐CD137L signalling stimulates inflammatory responses in AS development with pro‐inflammatory cytokines, such as IL‐6.^4^ However, the molecular mechanism underlying the induction of CD137‐producing Th17 cells remains largely unexplored.

IL‐6 has been observed to induce the improvement of Th17 cells and considered as a biomarker in the development and progression of inflammation during AS.^19^, ^20^ Therefore, blockade of IL‐6 attenuates the inflammatory responses in AS. IL‐6 contains several elements for binding of transcriptional factors, including AP‐1, NF‐КB and Camo‐responsive elements, and NF‐КB plays a crucial role in the induction of IL6 transcription.^21^ In this study, we observed that the expression of IL‐6 in exosomes was significantly increased after activation of CD137 signalling. We further investigated the molecular mechanism of CD137‐producing IL‐6 in ECs. Increasing evidence has linked CD137 and NF‐КB signalling in chronic inflammatory diseases,^4^, ^22^ and here we showed that activation of CD137 signalling could promote expression of IL‐6 in EC‐derived exosomes of ECs in a process mediated by NF‐КB. CD137‐CD137L interaction promotes a downstream signalling, NF‐КB, resulting in the increased expression of proinflammatory cytokine, IL‐6. CD137 signalling for IL‐6 is mediated by Akt phosphorylation and subsequent NF‐КB translocation, leading to the differentiation of Th17 cells.

Multiple tissues are involved in the AS development, especially the aorta. The changes in the composition of blood increase blood flow and fluid shear stress. ECs located in the intima layer which was in contact with blood flow of the aorta lumen. Once, ECs was induced to adopt to the changes in the pattern of blood flow, the fatty streak stage which was the initial stage of atherosclerosis started. ECs were responsible for the changes from hemodynamics, and the fluid shear stress per se induced exosomal IL‐6 released from ECs could be up‐taken by CD4 + T cells, promoting the differentiation of Th17 cells. The increased proportion of Th17 cells caused progression of endothelial dysfunction and amplified the inflammatory responses in arterial wall, which induced the development of atherosclerotic plaque. Then, EC‐derived exosomes release a higher level of IL‐6, which further induced peripheral inflammatory responses to trigger the differentiation of Th17 cells and promoted the formation of the atherosclerotic plaque. In this way, a malignant circle was created in AS. Therefore, blockade the malignant circle might alleviate the progression of the atherosclerotic plaque. To validate these hypotheses, ECs were treated with IL‐6 small‐interfering RNA, and then the CD4^+^T cells were exposed to EC‐derived exosomes. We found that the proportion of Th17 cells was markedly decreased, and the size of atherosclerotic plaque became smaller, suggesting that blockade of IL‐6 exerted a protective effect in AS.

The present study has three limitations. First, considering that the specific inhibitor of Akt or NF‐КB might induce other changes in exosomes, we only specifically blocked IL‐6, and it is necessary to elucidate complete identification of CD137‐CD137L signalling pathway in further studies. Second, because it is currently impossible to achieve the co‐culture of Th17 cells and ECs, and Th17 cells mainly secrete IL‐17, we explored the effect of IL‐17 on the function of ECs, which only partially explained the effect of Th17 cells on the function of ECs. Third, CD137‐CD137L interaction may promote some Micro‐RNA to activate the NF‐КB pathway in EC‐derived exosomes, so our results might not completely elucidate the potential mechanism underlying the inflammatory effect. And we will investigate the micro‐RNA profiling of the exosome fraction which might be useful in order to better characterize the molecular mechanisms underlying the inflammatory effect in our future studies.

**Figure 7:**
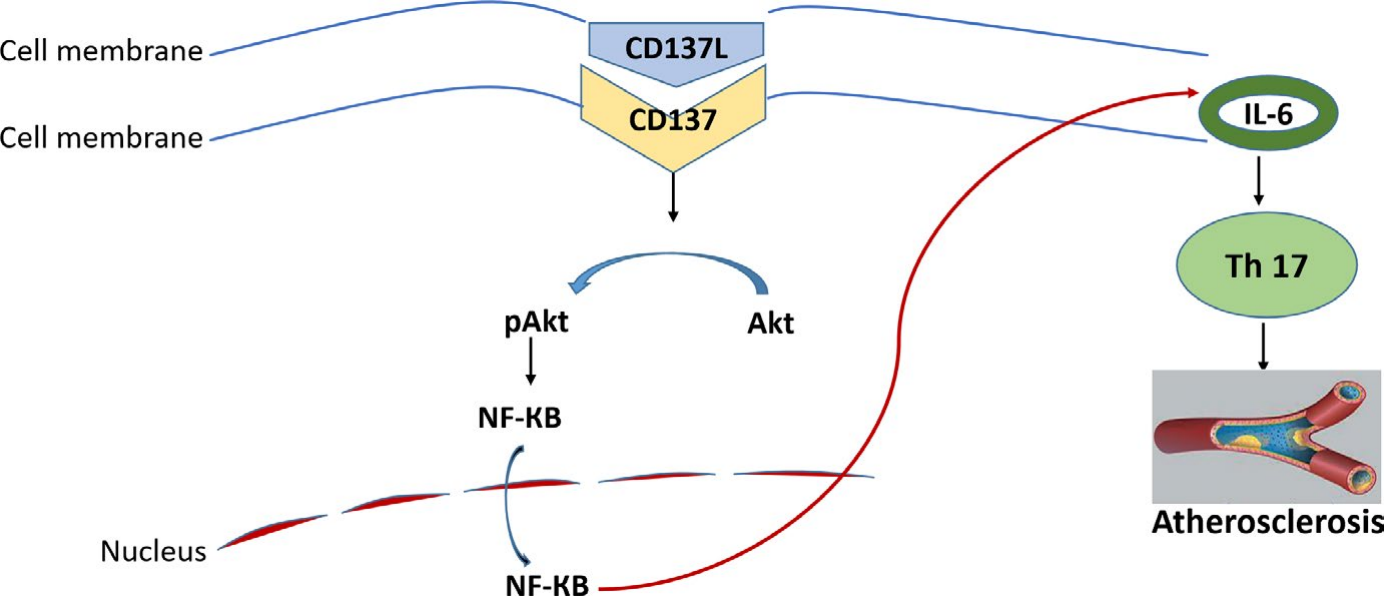
Schematic view of CD137 signalling pathway of amplifying Th17 generation in the progression of atherosclerosis. CD137‐CD137L interaction activates a downstream signalling pathway, resulting in the production of pro‐inflammatory cytokine, IL‐6. CD137 signalling for IL‐6 is regulated by Akt and NF‐КB. Th17 cell differentiation that induced by IL‐6 reaches a common downstream signalling pathway leading to atherosclerosis

In summary, we identified that EC‐derived exosomes functioned as a crucial molecule, which communicated local *vs*. peripheral inflammation in the AS development. ECs were involved in the accelerated inflammatory responses and AS partly because of the secreted exosomes of ECs, which regulated Th17 cell differentiation *via* IL‐6 expression mediated by NF‐КB pathway (Figure 7). These findings extended our knowledge on how EC‐derived exosomes affected the differentiation of Th17 cells and provided a potential method to prevent local and peripheral inflammation in AS.

## CONFLICT OF INTEREST

The authors declare that they have no conflict of interest.

## AUTHOR CONTRIBUTION

Hong Zhou and Liangjie Xu conceived the concept of the study. Tianxin Geng and Bo Li contributed to the design of the research and statistical analysis. Yi Liang and Liangjie Xu were involved in data collection. Liangjie Xu and Guangyao analysed the data. Jinchuan Yan co‐ordinated the project tasks. All authors edited, revised and approved the final version of the manuscript.

## Data Availability

All data included in this study are available upon request by contact with the corresponding author.
